# Ilizarov Versus AO External Fixator for the Treatment of Tibia Open Fractures

**Published:** 2011-12-01

**Authors:** S M Esmaeilnejad Ganji, M Bahrami, F Joukar

**Affiliations:** 1 Department of Orthopedics, Shahid Beheshti Hospital, Babol University of Medical Sciences, Babol, Iran

**Keywords:** Fracture, Tibia, Open, Ilizaro, AO external fixator, Treatment

## Abstract

**Background:**

In developing countries, Ilizarov or AO external fixator is usually used for treatment of tibial open fractures. The purpose of this study was to compare the efficacy of these two methods for treatment of tibial open fractures.

**Methods:**

From April 2002 to April 2010, 120 patients with open tibial fractures admitted to the Department of Orthopedics of Babol University of Medical Sciences entered this study. In each arm, 60 subjects randomly received Ilizarov or AO external fixator. All patients were followed at least for one year. These two groups were compared regarding non-union, malunion and cure rates.

**Results:**

The mean age of the patients in Ilizarov group was 32.35±11.28 and for AO were 31.3±10.99 years. Mean time for union in Ilizarov group was 5.25±1.85 and for AO external fixator was 5.85±2.13 months. Nonunion rate in Ilizarov group was 10% and for AO external fixator was 11.7%. Malunion rate in Ilizarov group was 10% and for AO external fixator was 18.3%. Totally, efficacy of treatment in the Ilizarov group was 81.7% and in AO external fixator was 65%.

**Conclusion:**

The efficacy of treatment in Ilizarov was higher than that AO external fixator in treatment of open tibial fractures.

## Introduction

Tibial fractures are the most common long bone fractures in the body.[1][2] Open fractures are common in this bone especially in the middle one third of its length.[2] Insufficient blood flow and lack of soft tissues in antero-medial aspect of tibia length predisposes tibia open fracture to non-union and development of infection.[3] Treatment of open tibial fractures has controversy among the orthopedics surgeons.[4] Currently, non-surgical procedures like using casts, brace or interventional attempts like inserting of plate, intramedullary nailing and external fixators are used for treatment of open tibial fractures.[5] Selection of any of the above methods are correlated with surgeon decision and economic status of patients. In North America, most of surgeons do reamed nailing for the treatment of open or closed tibial fractures.[5] In developing countries because of low facilities and lack of medical instruments, the selection of each method may differ.[6] Recently, external fixators like Ilizarov or AO external fixator are used extensively in developing countries but the rates of malunion and infection are relatively high.[3][7] With AO external fixator, the efficacy of treatment in two studies were reported to be 20- 31%.[8][9] This study was conducted to compare the efficacy of Ilizaro versus AO external fixator for treatment of tibial open fractures.

## Materials and Methods

This interventional study was conducted on patients with open tibial fractures admitted at the Emergency Department of Shahid Beheshti Hospital of Babol University of Medical Sciences from April 2002 to April 2010. Our department is the only orthopedics center and serves more than 1.7 million local residents in three large cities (Amol, Babol, and Babolsar) and the corresponding urban areas in the province of Mazandaran located across the Caspian Sea. Exclusion criteria were fractures involved articular surface, previous fracture of tibia, neurovascular damages, diabetic and immunocopromised patients. After admission, all cases were rehabilitated based on ATLS protocol.[10]

After taking x ray of the involved bone, antibiotic prophylaxis including cefazolin (2 g/8 hours) plus gentamicin (3-5 mg/kg/day) were administered and continued for 5 days. For contaminated wounds, penicillin was added to the mentioned regimen. Prophylaxis against tetanus was considered for all patients. [10][11] Observations and mechanisms for inducing of fractures were done. Physical examinations, assessment of neuro-vascular conditions in involved limb were carefully performed. Soft tissue injuries were classified based on Gustillo method. After stabilization, all cases X-rays were taken AP and lateral of total length of involved bone. After evaluation of the patients, all of them were transferred to operation room. After irrigation of the involved sites with 9 liters of normal saline and debridement, Ilizarov or AO external fixator was inserted. The Ilizarov or AO external fixator was done based on their guidelines.[2][12]

The primary end points of this study were lack of malunion and nonunion. The secondary endpoints included delayed union and infection. Malunion was defined as deformity of united bone with angulation> 5 degrees, shortening>1 cm and distal fragment rotation>15 degrees.13 Nonunion was defined when fractures were not developed union up to nine months after applying external fixator judged on clinically and radiologically. Delayed union was defined when fractures were not developed union up to 6 months judged on clinically and radiologically. Compliance was checked up at each visit during the course of treatment and was asked about the prescribed instruments. Efficacy (success rate) was defined as the rate of total cured cases in each arm. The sample size for each group was estimated to be 53 cases based on cure rate of 25% for AO external fixator and prediction of up to 6% for Ilizarov group.[8][9] For better accuracy, 60 cases were selected in each arm. The alpha and beta errors chosen for this calculation were 0.05 and 0.20, respectively. Patients admitted during the paired days received Ilizarov and the single days received AO external fixator until the sample sizes were completed. The study was approved by the research center of Babol Medical University and its Ethics Committee. All patients gave their written informed consent. Four days after procedures when patients had no infection or discharge and necrotized tissue and pain, they were ambulated and the weight bearing was encouraged as soon as possible for a range of painless or tolerable pain loading without taking any analgesic drug. Full weight bearing was permitted when there was not any disturbing pain in walking without crutch or any other aids. Every day, the sites of pins were irrigated with betadine and all cases were educated regarding caring of their pins sites. After discharging from the hospital, they were evaluated clinically and an x ray was obtained in all cases. This assessment was done each month for one year. When within three months of intervention healing did not progress precisely, we performed compression of Ilizarov or dynamization of AO external fixator. After union of the bone, fixators were removed and patellar tendon bearing casting or functional bracing was recommended for 6 weeks. Union of fracture was defined when painless weight bearing during walking occurred and x ray in AP and lateral views showed bridging calus in the 3 cortexes.5 If after six months, improvement process did not progress, it was named as delayed union and if 9 months (adding additional 3months) after intervention, repairing was not developed, it was called a nonunion.[2]

Bone deformity with angulation more than 5 degree, shortening more than one centimeter and rotational deformity>15 degree was considered as malunion. Malunion was measured by x ray of AP and lateral view of both legs for comparison. For determining the length of tibia, distance between proximal tibial surface, to the distal arthicular surface of tibia in two legs were measured. Determination of angulation between proximal and distal fragment of fracture was obtained by drowing 2 lines parallel to fragments longitudinal axis in two plane x-ray radiograms of involved leg and comparing it with conteralateral intact leg. Rotational deformity was determined by comparing axial CTscanogram of 2 ends points of tibias. Refracture was defined as the presence of fracture in previous fracture site in which consolidation occurred before the second trauma.[13] Infection of the pin sites was determined based on DAHL classification.[14] Data were analyzed by SPSS software (version 18, Chicago, IL, USA). The student t test was used to compare continuous variables and χ(2) test or Fisher Exact test was used to compare categorical variables. We used relative risk with 95% confidence interval to show all outcomes. Cox proportional hazards regression model (univariate and multivariate) was used to estimate the differences between these two methods of therapy after the adjustment of the baseline covariates like age, sex, Custillo classification of open fracture, pattern of fracture and kind of procedures. Differences with a p value of less than 0.05 were considered significant. All p values were 2-tailed.

## Results

The mean age of the patients in Ilizarov group was 32.35±11.28 (53 males, 7 females) and for AO fixator were 31.3±10.99 years (54 males and 6 females). Mean time for union in Ilizarov group was 5.25±1.85 and for AO external fixator was 5.85±2.13 months (p=0.1). Characteristics of the patients in the two treated groups were shown in Table 1. Risk factors for non-union and malunion were shown in Table 2. The rate of malunion in Ilizarove or AO external fixator were 10% and 18.3%, respectively (RR=0.49, 95%CI, 0.17-1.43, p=0.19). In Ilizarove group, for treatment of nonunion, intramedulary nailing (1 case), plaque (1 case) and iliac graft (2 cases) were used. In AO external fixator for treatment of nonunion, Ilizarov (3 cases), intramedulary nailing (2 cases), plaque and iliac graft (2 cases) were used. For treatment of malunion,operation was undertaken in two cases in Ilizarove group and in 6 cases in AO external fixator. All cases with infection of pin sites were treated with oral antibiotics. In AO external fixator, 3 cases were hospitalized for receiving of parenteral antibiotics and the rest of the patients were treated by oral antibiotics. Outcomes of treatment were shown in Table 3.

**Table 1:** Characteristics of patients in these two treated groups

**Variables**	**Ilizarov group No=60**	**AO group No=60**	***P* value**
Causes of fracture			
Motor or car accident, no (%)	52 (86.6)	57 (95)	NS^a^
Falling, no (%)	7(11.7)	2 (3.3)	NS
Gun shut no (%)	1 (1.7)	0 (0)	NS
Fighting, no (%)	0 (0)	1 (1.7)	NS
Gustillo classification of fracture			
Type I, no (%)	3 (5)	4 (6.7)	NS
Type II, no (%)	32 (53.3)	29 (48.3)	NS
Type IIIA, no (%)	19 (31.7)	21 (35)	NS
IIIB, no (%)	5 (8.3)	6 (10)	NS
IIIC	1 (1.7)	0 (0)	NS

^a^ not significant; The mean time for union in Ilizarov group was 5.25±1.85 months and for AO external fixator was 5.85±2.13 months (0.1).

**Table 2:** Risk factors for nonunion in the treatment of open tibial fractures

**Variable**	**HRb,d**	**95% CI^a^**	***P* value**	**HRc**	**95% CI**	***P* value**
Age	0.99	0.97-1.1	0.26	0.98	0.96-0.99	0.04
Gender	1.1	0.6-1.8	0.98	1.02	0.6-1.9	0.9
Gustillo	5.6	2.4-12.9	0.000	5.7	2.3-14.5	0.000
Fracture pattern	5.2	2.11-12.7	0.000	3.21	1.2-8.8	0.02
AO external fixator/ Ilizarov	1.31	0.9-1.92	0.15	1.47	0.98-2.21	0.06

^a^ CI, Confidence interval,

^b^ HR, Hazard ratio;

^c^ Results of univariate analysis;

^d^ Results of multivariate analysis.

**Table 3:** Outcome of treatment with Ilizarov or AO external fixature in patients with tibial open fractures

**Outcome**	**Ilizarov group No (%)**	**AO external fixator No (%)**	**RR a**	**95% CI b**	***P* value**
Nonunion	4 (6.7)	7 (11.7)	0.54	0.15-1.95	0.34
Malunion	6 (10)	12 (20)	0.44	0.15-1.27	0.12
Referacture	1 (1.7)	2 (3.3)	0.49	0.043-5.57	1
Failure rate	11 (18.3)	21 (35)	0.41	0.18-0.96	0.039
Cure rate	49 (81.7)	39 (65)	2.39	1.03-5.56	0.039

^a^ RR; relative risk,

^b^ CI; confidence interval

## Discussion

In this study, no differences were found regarding the mean time for union, malunion and refracture when we used Ilizarove or AO external fixator for the treatment of open tibia fractures. Wani et al. and Hosney et al. found similar mean time for union of fractures that were 6 and 5.6 months respectively when they used Ilizarov for treatment of tibial open fractures and were similar to that found in our study.[6][15] Sen et al. found longer duration of time for union of fractures (7.5 months) with Ilizarov and was higher than that we found in this study. In their study, all patients had Gustillo III fractures with mean bone loss of 5 centimeters and 2.5×3.5 centimeters soft tissue loss with extension of fractures to adjacent articular space.[16] Qureshi et al. reported that 3.3% of their patients who were treated with Ilizarov had nonunion which was lower than the results of our study. In their study, both open and closed tibial fractures were included in the study and those who needed flap were excluded.[17] Ocguder et al. reported the rate of delayed union to 15.5% when they used Ilizarov and was longer than our findings. The reason for delayed union in their study was insufficient fixation of the fractures.[18] Wani et al. reported the rate of malunion to 10% when they treated open tibial fractures with Ilizarov and was similar to our findings.[6]

Inan et al. reported the rate of malunion with Ilizarov to 21.5% which was higher than our results. Angulation of more than 7 degree and shortening of the limb were their complications.[19] Infection of the pin sites was seen in 27.4% of patients treated in Ilizarov group and was similar to our findings.[18] In two studies, the mean time for union of tibial fractures using AO external fixator was reported to be 21 to 36.9 weeks and was longer than our results.[20][21] In these two studies, only fractures of Gustillo III were selected in their studies and most of their patients were too old to be ambulated early. In our studies, all cases were ambulated within 3-4 days after procedures. Another study performed in Gustillo fracture III with AO external fixator, delayed union was noticed to be 40% and was more than the results obtained by our study.[19] With AO external fixator, another study showed malunion to be 31%.[10] Henly et al. reported that delayed union or nonunion were related with extensive soft tissues damages.[8] Papaioannov showed nonunion in 20% of their patients when treated with AO external fixator. They also showed that the rates of nonunion with Gustillo II and III when compared with Gustillo I and lost fractures were higher.[9] In this study, we found better efficacy of Ilizarov in comparison to AO fixator for the treatment of tibia open fractures (Table 3). We think that control of fracture fragments by Ilizarov frame was better than AO external fixator because of three dimensional manipulations of fragments possibility at the operation scene, and better achievement of compression in fracture site during post operation management.

In conclusion, the results of our study showed that the efficacies of treatment with Ilizarov was higher than AO external fixator in treatment of tibia open fractures.
